# Growth differentiation factor-15: a p53- and demethylation-upregulating gene represses cell proliferation, invasion, and tumorigenesis in bladder carcinoma cells

**DOI:** 10.1038/srep12870

**Published:** 2015-08-07

**Authors:** Ke-Hung Tsui, Shu-Yuan Hsu, Li-Chuan Chung, Yu-Hsiang Lin, Tsui-Hsia Feng, Tzu-Yi Lee, Phei-Lang Chang, Horng-Heng Juang

**Affiliations:** 1 Department of Urology, Chang Gung Memorial Hospital, Kwei-Shan, Tao-Yuan, Taiwan, ROC; 2 Department of Anatomy, College of Medicine, Chang Gung University, Kwei-Shan, Tao-Yuan, Taiwan, ROC; 3 Department of Traditional Chinese Medicine, College of Medicine, Chang Gung University, Kwei-Shan, Tao-Yuan, Taiwan, ROC; 4 School of Nursing, College of Medicine, Chang Gung University, Kwei-Shan, Tao-Yuan, Taiwan, ROC

## Abstract

Growth differentiation factor-15 (GDF15), a member of the TGF-β superfamily, affects tumor biology of certain cancers, but remains poorly understood in bladder cancer cells. This study determined the expression, regulation, function, and potential downstream target genes of GDF15 in bladder carcinoma cells. The transitional papilloma carcionoma cells (RT4) expressed higher levels of GDF15 as compared with the bladder carcinoma cells (HT1376 and T24). Treatments of recombinant human GDF15 (rhGDF15) reduced the proliferations of HT1376 and T24 cells. Expression of GDF15 was upregulated via DNA demethylation and p53. The cell proliferation, invasion, and tumorigenesis were reduced in ectopic overexpression of GDF15, while enhanced in GDF15 knockdown. The expressions of mammary serine protease inhibitor (MASPIN) and N-myc downstream-regulated family genes (NDRG1, NDRG2, and NDRG3) were upregulated by GDF15 overexpressions and rhGDF15 treatments in bladder carcinoma cells. GDF15 knockdown induced epithelial-mesenchymal transition (EMT) and F-actin polarization in HT1376 cells. Our results suggest that enhanced expressions of MASPIN and N-myc downstream-regulated family genes and the modulation of EMT may account for the inhibitory functions of GDF15 in the cell proliferation, invasion, and tumorigenesis of bladder carcinoma cells. The GDF15 should be considered as a tumor suppressor in human bladder carcinoma cells.

Urinary bladder carcinoma is the fourth leading malignancy in American males and the eighth most common cause of malignancy-related death^1^. Approximately 20% to 25% of primary bladder cancers have invaded the muscle layer of the bladder wall by the time they were diagnosed, and thus suggesting a poor prognosis; in addition, seventy percent of papillary and superficial tumors recur within two years of surgical excision^2^. Because the effective strategies for early detection of bladder cancer remain elusive, the recurrence and mortality rates are high even though the risk factors of bladder cancer have been identified^3^,^4^,^5^. Thus, it is practical to explore a new biomarker in detection and develop an understanding in the molecular mechanism of the target gene for bladder cancer.

Growth differentiation factor-15 (GDF15) is a secretory dimeric protein that possesses characteristic structures of cytokines in the TGF-β superfamily. GDF15 is also known as PLAB (placental bone morphogenetic protein), PTGF-β (placental transforming growth factor-β), NAG-1 (nonsteroidal anti-inflammatory drug-activated gene-1), and PDF (prostate differentiation factor)^6^. Previous studies have indicated divergent effects of GDF15 in brain, ovarian, intestinal, prostate, and hepatocellular carcinoma^7^,^8^,^9^,^10^,^11^,^12^,^13^ suggesting that function of GDF15 has a diverse range of tissue-specific and cell-specific presentations^14^,^15^,^16^. The expression, function, and regulation of GDF15 in bladder cancer have not been fully explored although two recent reports indicated that the epigenetic modulation of GDF15 is an important biomarker in the bladder cancer and the upper tract urothelial carcinoma^17^,^18^.

The objectives of this study were to determine the expression and regulation of GDF15 in human bladder carcinoma cells, to investigate the tumorigenesis and invasiveness in bladder carcinoma cells engineered to overexpress or knockdown GDF15, and to evaluate the potential mechanisms by which GDF15 suppresses tumorigenesis in human bladder carcinoma cells.

## Results

### Expression of GDF15 in bladder cell lines

**Figure 1:**
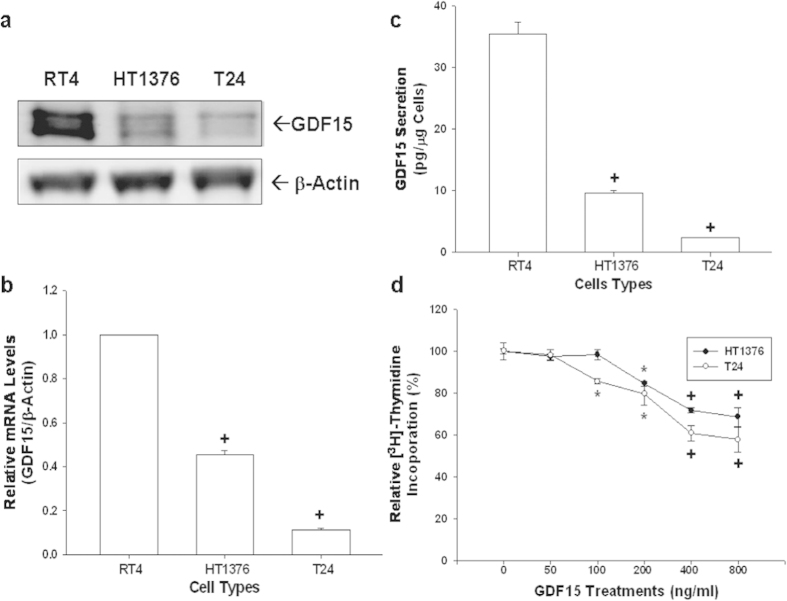
Gene expressions of GDF15 in human bladder carcinoma cells and the effect of GDF15 on cell proliferation. All bladder cells used in this study were serum starved for 24 hours subsequently incubated in RPMI media containing 10% FCS for another 24 hours. (**a**) Cell proteins were then lysed for immunoblotting assay. (**b**) Total RNA was extracted from cells for the RT-qPCR assay. Data are presented as mean-fold (±S.E.; n = 3) in relation to that of the RT4 cell group. (**c**) Conditioned media was collected for ELISA in order to determine the level of GDF15 secretion in the various bladder carcinoma cells. Data is presented as the mean (±S.E.; n = 6) of the GDF15 levels. (**d**) Proliferation rates in HT1376 (black circle) and T24 (white circle) cells treated with various concentrations of GDF15 were determined by ^3^H-thymidine incorporation assays. Each point on the curve represents the mean-percentage (±S.E.; n = 6) relative to solvent-treated group (*P < 0.05, ^+^P < 0.01).

The protein levels of GDF15 of three cultured bladder cell lines (RT4, HT1376, and T24) were assessed using immunoblotting assays (Fig. 1a). The transitional papilloma cells (RT4) expressed higher levels of GDF15 as compared with metastatic bladder carcinoma cells (HT1376 and T24). Results of RT-qPCR (Fig. 1b) indicated that levels of GDF15 mRNA were approximately 9-fold and 2-fold higher in RT4 cells as compared to T24 and HT1376 cells, respectively. GDF15 secretion levels determined by ELISA analysis yielded similar results (Fig. 1c).

### GDF15 decreased cell proliferation in human bladder carcinoma cells

To investigate the role of GDF15 in bladder carcinoma cells, we treated two human bladder cancer cell lines, HT1376 and T24 cells, with recombinant human GDF15 protein (rhGDF15). As shown in Fig. 1d, rhGDF15 attenuated cell proliferation of both HT1376 and T24 cells. Results indicated that cell proliferation decreased 31% and 42%, respectively, when HT1376 and T24 cells were treated with 800 ng/ml of rhGDF15 for 48 hours.

### p53 and demethylation enhance expressions of GDF15 in human bladder carcinoma cells

**Figure 2:**
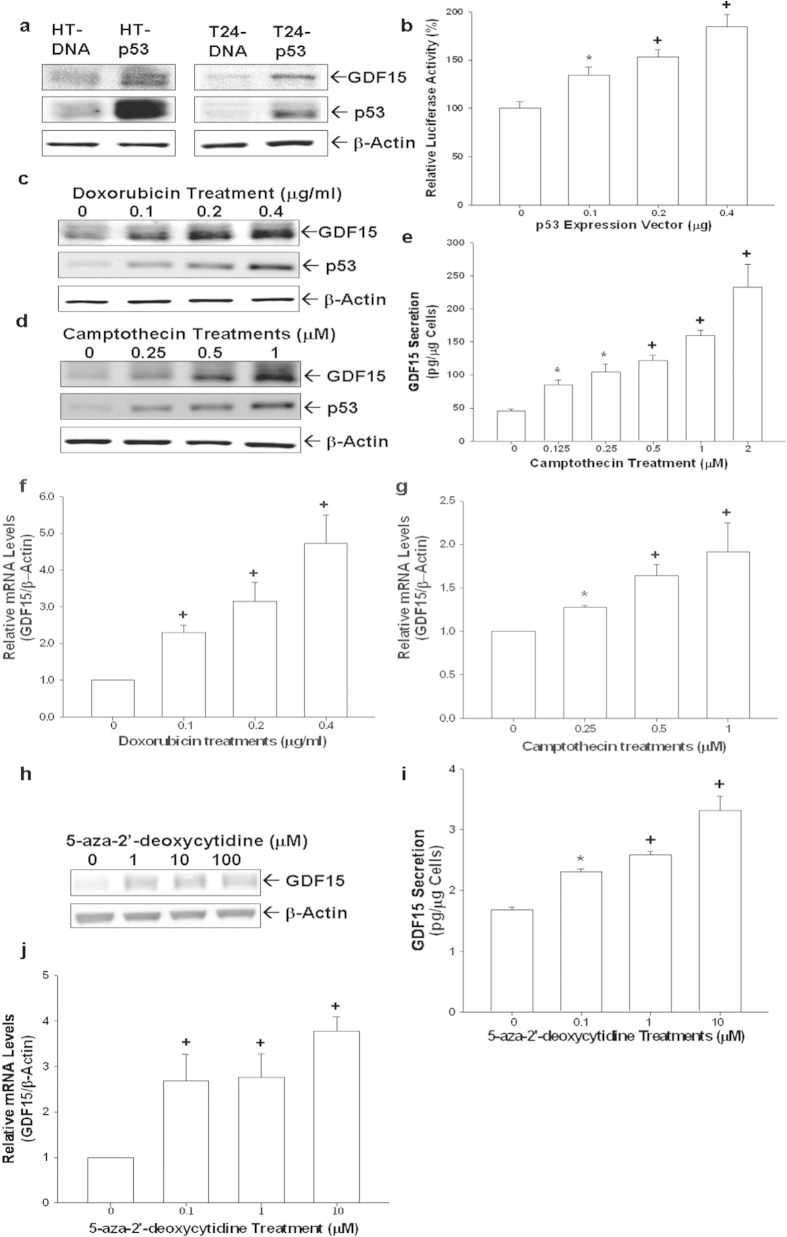
Modulation of p53 and demethylation on GDF15 expression in human bladder carcinoma cells. (**a**) HT1376 (left) and T24 (right) cells were transiently overexpressed with p53 for 72 hours. The levels of GDF15 and p53 expressions were determined by immunoblotting assays. (**b**) The GDF15 report vector was co-transfected with various concentrations of p53 expression vector into HT1376 cells for 72 hours. Data are expressed as the mean percentage ± S.E. (n = 6) of luciferase activity relative to mock-transfected groups. Expressions of GDF15 and p53 in RT4 cells following doxorubicin (**c**) or camptothecin (**d**) treatments were determined by immunoblotting assays. (**e**) GDF15 secretion in RT4 cells following camptothecin treatments was determined by ELISA. Data are expressed as mean (±S.E.; n = 6) of the GDF15 levels. Total RNAs were extracted from doxorubicin treated (**f**) and camptothecin treated RT4 (**g**) cells for RT-qPCR assays. T24 cells were treated with various concentrations of 5-Aza-2′-deoxycytidine for 48 hours and then GDF15 expression was determined by immunoblotting (**h**), ELISA (**i**), and RT-qPCR assays (**j**). Data are expressed as the mean-fold ± S.E. (n = 3) relative to solvent-treated groups and mean (±S.E.; n = 6) of the GDF15 levels. (*P < 0.05, ^+^P < 0.01).

The expressions of p53 and GDF15 in transient p53-overexpressed HT1376 (HT-p53) and T24 (T24-p53) cells were determined by immunoblotting assays. Results indicated that p53 induced GDF15 expressions in HT1376 (p53-mutant) and T24 (p53-null) cells (Fig. 2a). Similar results were also shown in the transient gene expression assays (Fig. 2b). To further verify the correlation between p53 and GDF15, we treated RT4 (p53 wild-type) cells with doxorubicin and camptothecin. Results showed that both doxorubicin and camptothecin induced expressions of p53 and GDF15 in RT4 cells in a dose-dependent manner (Fig. 2c,d). Similar results were obtained using ELISA (Fig. 2e) and RT-qPCR analyses (Fig. 2f,g). Further immunoblotting assays demonstrated that knockdown of GDF15 did not affect p53 expressions in RT4 cells suggesting p53 was not the downstream gene of GDF15 in bladder carcinoma cells (Figure supplement 1). In order to evaluate the effect of demethylation on GDF15 expression, we treated T24 cells with 5-aza-2′-deoxycytidine, an inhibitor of DNA methylation. Results of immunoblotting (Fig. 2h), ELISA (Fig. 2i), and RT-qPCR (Fig. 2j) assays indicated that expression of GDF15 in T24 cells increased dose-dependently at the presence of 5-aza-2′-deoxycytidine.

### Overexpression of GDF15 inhibits cell proliferation, invasion, and tumorigenesis of HT1376 cells

**Figure 3:**
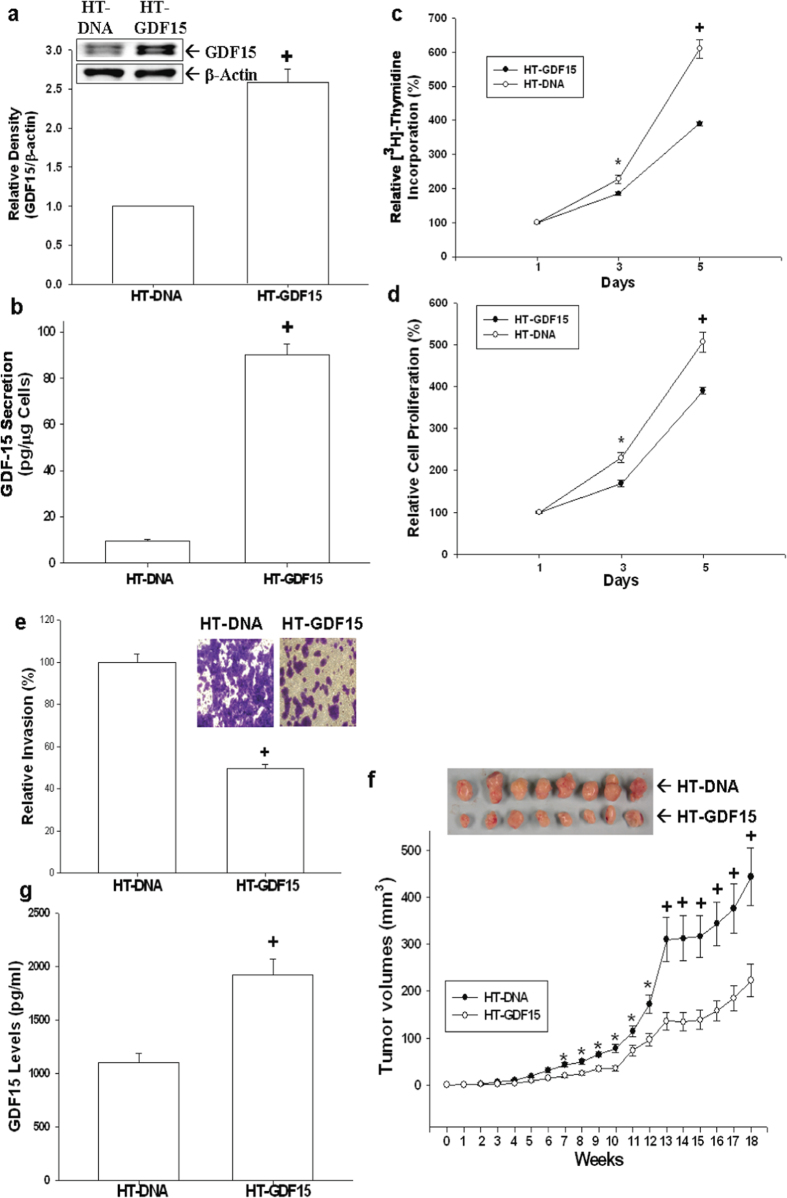
Effect of GDF15 overexpressed on cell proliferation, invasion, and tumorigenesis in HT1376 cells. Expressions of GDF15 in HT1376 cells stably transfected with pcDNA3 (HT-DNA) or pcDNA-GDF15 (HT-GDF15) expression vector were determined by immunoblotting assays (**a**) and ELISA (**b**). Data are expressed as mean-fold (±S.E.; n = 3) in relation to the HT-DNA cell group and the mean (±S.E.; n = 6) of the GDF15 levels. Proliferations of HT-DNA (white circle) and HT-GDF15 (black circle) cells were determined according to the incorporation of ^3^H-thymidine (**c**) and MTS assays (**d**). Each point on the curve represents the mean-percentage (±S.E.; n = 6) of that on day 1. (**e**) The invasive ability of cells was determin**e**d by *in vitro* matrigel invasion assays. Data are presented as mean-percentage (±S.E.; n = 3) in relation to that of the HT-DNA cell group. (**f**) Nude mice were inoculated subcutaneously with HT-DNA (black circle) or HT-GDF15 (white circle) cells. At the indicated days, tumor size was measured using vernier calipers, and results are presented as tumor size in mm^3^ (±S.E.). (**g**) Blood samples were collected from experimental animals by cardiocentesis immediately after sacrificed, and GDF15 levels were determined by ELISA. Data is presented as mean (±S.E.; n = 6) of the GDF15 levels. (*P < 0.05, ^+^P < 0.01).

Human GDF15 expression vector was transfected in bladder carcinoma HT1376 cells to evaluate the function of native GDF15 with regard to cell proliferation and invasion. Results of immunoblotting assays (Fig. 3a) and ELISA (Fig. 3b) confirmed that GDF15-overexpressed HT1376 (HT-GDF15) cells exhibited greater GDF15 protein levels as compared to mock-transfected (HT-DNA) cells. *In vitro* studies using ^3^H-thymidine incorporation assays revealed that mock-transfected HT1376 (HT-DNA) cells increased in cell proliferation by 6.15-fold after 5-day incubation period; however, the cell proliferation of GDF15-transfected (HT-GDF15) cells increased only by 3.87-fold. This suggested that stable overexpression of GDF15 in HT1376 cells attenuated cell proliferation (Fig. 3c). Similar results were also found in MTS assays (Fig. 3d). Results of *in vitro* matrigel invasion assays indicated that the invasive ability of HT-GDF15 cells was approximately 2-fold lower than that of HT-DNA cells (Fig. 3e). The effect of GDF15 on the growth of tumors *in vivo* was evaluated using xenografts in nude mice. Tumors generated from HT-DNA cells grew faster than those from HT-GDF15 cells. Additionally, tumors derived from HT-GDF15 cells were only about 50% size of the tumors generated from HT-DNA cells (222.61 ± 34.89 vs. 443.24 ± 61.73 mm^3^) after 18 weeks of growth (Fig. 3f). ELISA assays of serum samples collected from experimental animals by cardiocentesis revealed that GDF15 levels in the circulatory system were approximately 1.75 times higher in animals inoculated with HT1376-GDF15 cells than in animals inoculated with HT-DNA cells (Fig. 3g).

### GDF15-knockdown enhances cell proliferation, invasion, and tumorigenesis of HT1376 cells

**Figure 4:**
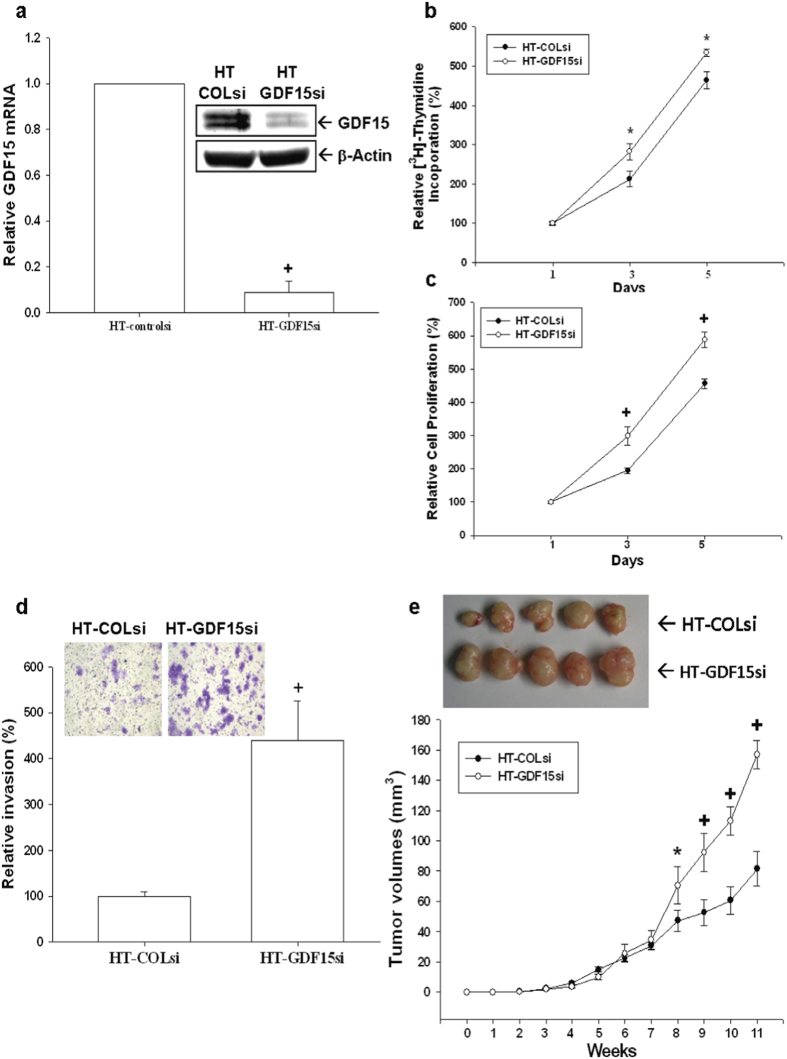
Knockdown of GDF15 enhances cell proliferation and invasion in human bladder carcinoma HT1376 cells. Expressions of GDF15 in mock-knockdown HT1376 (HT-COLsi) and GDF15 knockdown HT1376 (HT-GDF15si) cells were determined by immunoblotting (**a**, top) and RT-qPCR (**a**, bottom) assays. Data are expressed as mean-fold of the GDF15 levels (±S.E.; n = 3) in relation to the HT-COLsi cell group. Proliferations of HT-GDF15si (white circle) and HT-COLsi (black circle) cells were determined according to the incorporation of ^3^H-thymidine (**b**) and MTS assays (**c**). Each point on the curve represents the mean-percentage (±S.E.; n = 6) of that on day 1. (**d**) Invasive ability of cells was determined by the *in vitro* matrigel invasion assays. Data are presented as mean-percentage (±S.E.) in relation to the HT-COLsi cell group. (**e**) Nude mice were inoculated subcutaneously with HT-COLsi (black circle) or HT-GDF15si (white circle) cells. Tumor size as measured using vernier calipers. Results are presented as tumor size in mm^3^ (±S.E.), which measured at the indicated time intervals. (*P < 0.05, ^+^P < 0.01).

To reconfirm the function of GDF15 in bladder carcinoma cells, we knocked down GDF15 in HT1376 cells. The expressions of GDF15 in GDF15-knockdown HT1376 (HT-GDF15si) cells and mock-knockdown (HT-COLsi) cells were determined by immunoblotting and RT-qPCR assays (Fig. 4a). Results of ^3^H-thymidine incorporation (Fig. 4b) and MTS (Fig. 4c) assays revealed that cell proliferation was enhanced when GDF15 was knocked down in HT1376 cells. Results of *in vitro* matrigel invasion assays indicated that the invasive ability of HT-GDF15si cells was approximately 4.5-fold higher than that of HT-COLsi cells (Fig. 4d). Results of *in vivo* xenografts animal study showed that tumors generated from HT-GDF15si cells grew faster than those from HT-COLsi cells. After 11 weeks of growth, tumors derived from HT-COLsi cells were only approximately 52% of the size (81.45 ± 11.47 vs. 157.01 ± 9.46 mm^3^) of tumor generated from HT-GDF15si cells (Fig. 4e).

### Overexpression of GDF15 inhibits cell proliferation and invasion of T24 cells

**Figure 5:**
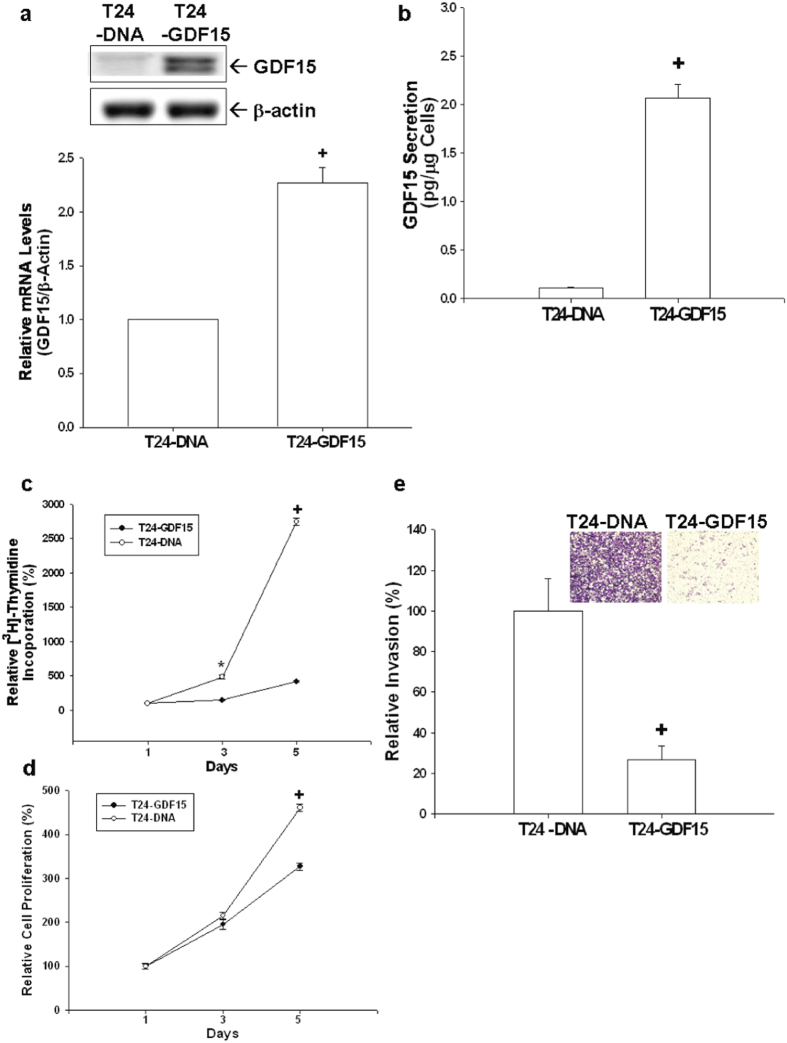
Effects of GDF15 overexpressed on cell proliferation and invasion in T24 cells. Expressions of GDF15 in T24 cells transfected with pcDNA3 (T24-DNA) or pcDNA-GDF15 (T24-GDF15) expression vector were determined by immunoblotting assays (**a**, top), RT-qPCR assays (**a**, bottom) and ELISA (**b**). Data are expressed as mean-fold (±S.E.; n = 3) in relation to the T24-DNA cell group and the mean (±S.E.; n = 6) of the GDF15 levels. Cell proliferations in T24-DNA (white circle) and T24-GDF15 (black circle) were determined according to ^3^H-thymidine incorporation (**c**) and MTS assays (**d**). Each point on the curve represents the mean-percentage (±S.E.; n = 6) of that on day 1. (**e**) The invasive ability of cells was determined by the *in vitro* matrigel invasion assays. Data are presented as the mean-percentage (±S.E.) in relation to that of the T24-DNA cell group. (*P < 0.05, ^+^P < 0.01).

GDF15 overexpression also inhibits cell proliferation and invasion in T24 cells. Results of immunoblotting (Fig. 5a, top), RT-qPCR (Fig. 5a, bottom), and ELISA (Fig. 5b) assays confirmed that GDF15-overexpressed T24 cells (T24-GDF15) exhibited greater GDF15 protein levels as compared to mock-transfected T24 (T24-DNA) cells. Results from ^3^H-thymidine incorporation assays revealed that mock-transfected T24 (T24-DNA) cells showed a 28-fold increase in numbers after 5-day incubation period; however, the numbers of GDF15-transfected (T24-GDF15) cells increased only by 3-fold (Fig. 5c). Results determined by MTS assays showed similar results (Fig. 5d). *In vitro* matrigel invasion assays revealed that the invasive ability of T24-GDF15 cells was significantly decreased as compared to that of T24-DNA cells (Fig. 5e).

### GDF15 modulates the expressions of MASPIN, NDRG1, and NDRG3 genes in bladder carcinoma cells

**Figure 6:**
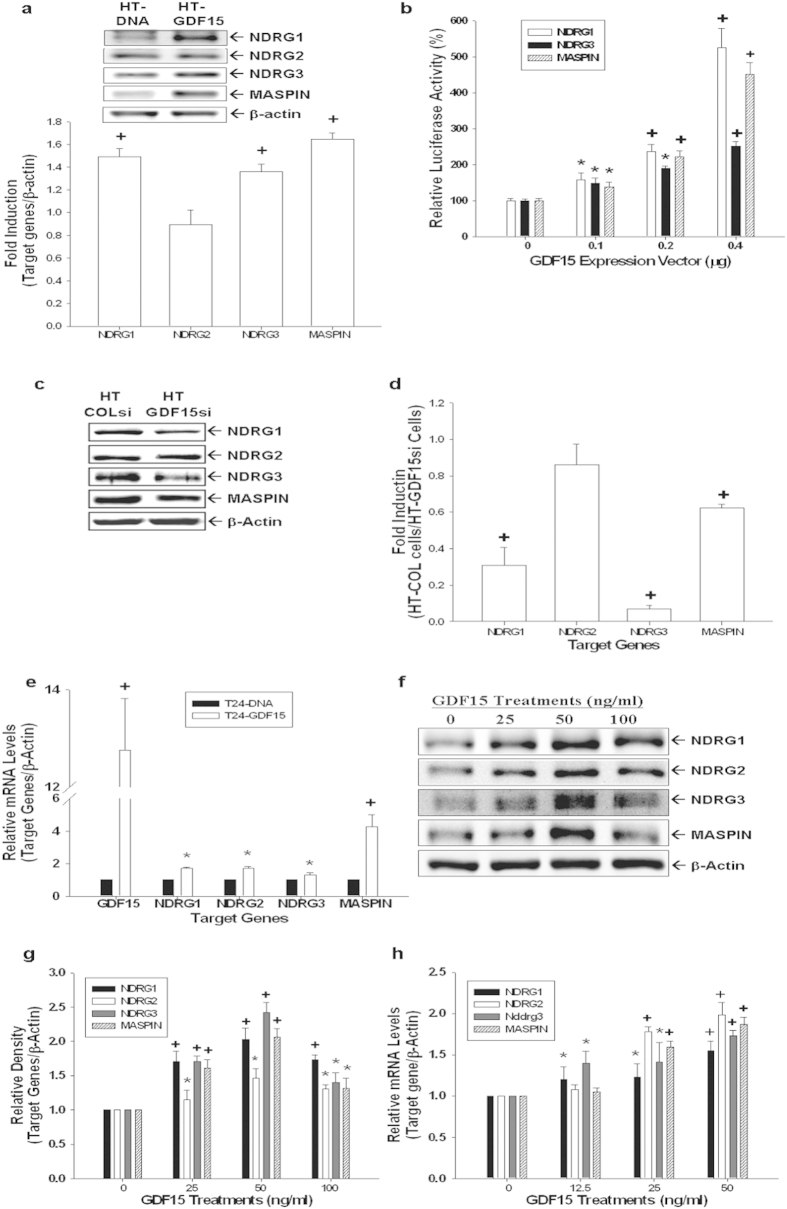
Modulation of GDF15 on the expressions of MASPIN, NDRG1, NDRG2, and NDRG3 genes in bladder carcinoma cells. (**a**) Differences in expressions of NDRG1, NDRG2, NDRG3, and MASPIN between HT-DNA and HT-GDF15 cells were determined by immunoblotting assays. Data of quantitative analysis are expressed as the intensity of the protein bands produced from the target gene/β-actin (±S.E.; n = 3) of HT-GDF15 cells relative to that of the HT-DNA group. (**b**) The reporter vectors of NDRG1, NDRG3, and MASPIN were cotransfected with various concentrations of GDF15 expression vectors into HT1376 cells for 72 hours. Data are expressed as the mean percentage ± S.E. (n = 6) relative to the mock-transfected groups. Expressions of GDF15, NDRG1, NDRG2, NDRG3, and MASPIN in mock-knockdown HT1376 (HT-COLsi) and GDF15-knockdwon HT1376 (HT-GDF15si) cells were determined by immunoblotting (**c**) and RT-qPCR (**d**) assays. Data are presented as mean-fold (±S.E.) in relation to the HT-COLsi cell group. (**e**) Differences in the expressions of GDF15, NDRG1, NDRG2, NDRG3, and MASPIN genes between T24-DNA and T24-GDF15 cells were determined by RT-qPCR assays. Data are presented as mean-fold (±S.E.) in relation to the T24-DNA cell group. Differences in the expressions of NDRG1, NDRG2, NDRG3, and MASPIN genes following treatments with various concentrations of rhGDF15 were determined by immunoblotting (**f**) and RT-qPCR (**h**) assays. Data of quantitative analysis are expressed as the intensity of the protein bands produced from the target gene/β-actin (±S.E.; n = 3) relative to that of the solvent-control group (**g**). (*P < 0.05, ^+^P < 0.01).

Results of immunoblotting assays revealed that ectopic-overexpression of GDF15 in HT1376 cells enhanced protein expressions of NDRG1, NDRG3, and MASPIN genes; however, GDF15-overexpressed did not affect the expression of NDRG2 in HT1376 cells (Fig. 6a). Similar results also found in the reporter assays which showed that transient overexpression of GDF15 enhanced the promoter activities of NDRG1, NDRG3, and MASPIN reporter vectors (Fig. 6b). Further results indicated that expressions of NDRG1, NDRG3, and MASPIN genes in GDF15-knockdown (HT-GDF15si) cells were depressed in comparison to mock-knockdown (HT-COLsi) cells, which were determined by immuoblotting (Fig. 6c) and RT-qPCR (Fig. 6d) analyses. Results of RT-qPCR (Fig. 6e) assays revealed that ectopic-overexpression of GDF15 upregulated the expressions of NDRG1, NDRG2, NDRG3, and MASPIN genes in T24 cells. Furthermore, rhGDF15 treatments enhanced gene expressions of NDRG1, NDRG2, NDRG3, and MASPIN in T24 cells as determined by immunoblotting (Fig. 6f,g) and RT-qPCR (Fig. 6h) assays.

### GDF15 modulates EMT markers and F-actin polarization in HT1376 cells

**Figure 7:**
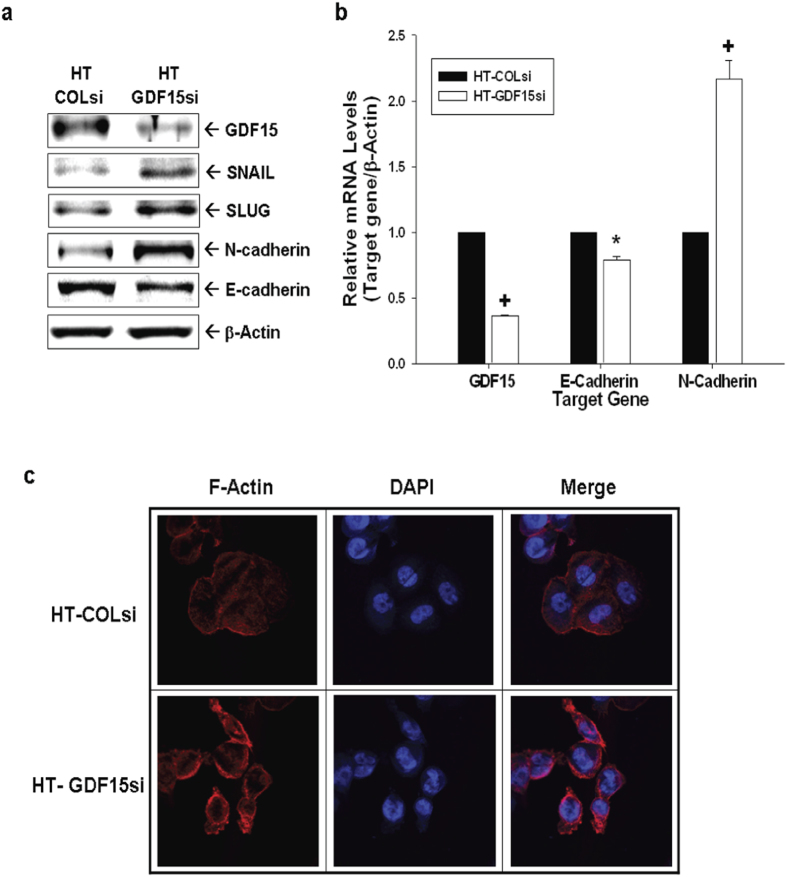
Expression of GDF15 modulates the expression of epithelial-mesenchymal transition markers in human HT1376 cells. (**a**) Expression levels of SNAIL, SLUG, E-cadherin, and N-cadherin in HT-GDF15si and HT-COLsi cells were determined by immunoblotting **a**ssays. (**b**) Expressions of GDF15, E-cadherin, and N-cadherin in HT-GDF15si (white bars) and HT-COLsi (black bars) cells were determined by RT-qPCR assays. Data are presented as mean-fold (±S.E.) in relation to the HT-COLsi cell group. (**c**) Distribution of F-actin (red) between HT-GDF15si and HT-COLsi cells was determined by immunofluorescence staining. DAPI (blue) was applied to stain the nucleus. (*P < 0.05, ^+^P < 0.01).

We continued to evaluate the modulation of GDF15 on EMT markers and F-actin polarization in HT1376 cells. As shown in Fig. 7a, GDF15-knockdown (HT-GDF15si) cells expressed lower levels of E-cadherin and higher N-cadherin, SNAIL, and SLUG than those in mock-knockdown (HT-COLsi) cells. RT-qPCR analyses indicated that E-cadherin expression in HT-GDF15si cells decreased by 20% while N-cadherin expression showed a 2.2-fold increase compared with that of HT-COLsi cells (Fig. 7b). Results of immunofluorescence staining showed that HT-GDF15si cells have more prominent F-actin expression within the cytoplasm and polar distribution as compared to HT-COLsi cells, indicating GDF15 knockdown increased F-actin polarization of HT1376 cells.

## Discussion

Studies have shown that the pleiotropic action of GDF15 is involved in cell growth inhibition, apoptosis induction, and invasion enhancement in various cancer cell lines^19^. The divergent effects of GDF15 work not only highly tissue-specific but also cell-specifically^7^,^14^,^15^,^16^. Moreover, the expression, function, and regulation of GDF15 in bladder cancer have not been fully elucidated. Recent DNA-based analysis revealed that the promoter methylation status of GDF15 gene was significantly higher in urine sediments and tissue samples from patients with bladder cancer or upper tract urothelial carcinoma, indicating that epigenetic modulation of GDF15 is an important biomarker in these diseases^17^,^18^. In combination of the results in present *in vitro* study revealed that GDF15 expression was lower in the bladder carcinoma cells, HT1376 and T24, as compared to the RT4 cells which derived from explants of a recurrent papillary bladder tumor, suggesting that GDF15 may be associated with bladder neoplasia. However, further investigation is needed to evaluate whether the levels of GDF15 in the urine or the bladder tissues can be used as the tumor marker for bladder cancer.

Ectopic overexpression of p53 in T24 and HT1376 cells, the p53 null and p53 mutant, induced expression of GDF15; moreover, p53 expressions induced by doxorubicin or camptothecin in p53 wild-type RT4 cells in turn induced the GDF15 expressions at transcriptional and translational levels. These results clearly demonstrated that p53 upregulates GDF15 expression in bladder cancer cells, and are in agreement with recent studies of cancer cells from other tissues, such as prostate, head and neck, and breast, in which suggested that GDF15 is a p53-downstream gene^7^,^20^,^21^,^22^. However, knockdown of GDF15 did not affect significantly on the p53 expression in RT4 cells indicating no feedback loop between p53 and GDF15.

Following demethylation by treatments of 5-aza-2′-deoxycytidine, our results indicated that GDF15 mRNA and protein levels in T24 cells increased in a dose-dependent manner. The DNA methylation is usually associated with the loss of gene expression^23^. Thus, based on our results and recent findings reported that GDF15 is hypermethylated in urine sediments of patient with bladder cancer^17^, we suggested that loss of the GDF15 expression by hypermethylation may play a critical role in bladder neoplasia.

Results in this study revealed that rhGDF15 treatments or ectopic overexpression of GDF15 downregulated cell proliferation of T24 and HT1376 cells *in vitro*. Moreover, GDF15-overexpressed retarded tumor growth in xenograft animal studies, while GDF15-knockdown enhanced tumor growth. Our findings indicated that GDF15 attenuated cell growth of bladder carcinoma cells *in vitro* and *in vivo*. Further results of matrigel invasion assays revealed that GDF15 also affects the cell invasion. Overexpression of GDF15 downregulated cell invasion in HT1376 and T24 cells, while GDF15-knockdown enhanced cell invasion in HT1376 cells. Taken together, our results clearly demonstrated that GDF15 has anti-proliferation and anti-invasion functions in bladder carcinoma cells.

We evaluated the potential downstream target genes of GDF15 in bladder carcinoma cells. Results of immunoblotting, RT-qPCR, and reporter assays showed that expression of MASPIN was upregulated in cases where GDF15 was ectopic-overexpressed in HT1376. Conversely, GDF15-konckdown decreased the expression of MASPIN. In addition, the upregulation of MASPIN was observed in GDF15-overexpressed T24 cells or rhGDF15-treated T24 cells. Previous reports indicated that expression of MASPIN, a mammary serine protease inhibitor with tumor suppressing properties, was inversely correlated with the patient prognosis and the recurrence of bladder cancer^24^,^25^. This is the first study to demonstrate that MASPIN is the downstream target gene of GDF15 although the precise molecular mechanism responsible for the effect of GDF15 on the MASPIN still needs to be further investigated.

The N-myc downstream-regulated gene (NDRG) family of proteins consists of 4 members (NDRG1, NDRG2, NDRG3, and NDRG4) which contribute to the cell proliferation, differentiation, development, and stress response^26^. Although the function and expression of NDRG family genes with regard to bladder carcinoma cells remain poorly understood, our previous report has indicated that NDRG1 is upregulated by interleukin 6, which blocks cell proliferation and invasion in bladder carcinoma cells^27^. Further, a previous study found that NDRG2 expression is negatively correlated with pathologic stages, and NDRG2-overexpressed downregulates the proliferation and invasion of bladder carcinoma cells *in vitro* and *in vivo*^28^. Nowadays, no report has yet described the expression or regulation of NDRG3 in bladder cancer. Our study is the first study to demonstrate the modulations of NDRG1, NDRG2, and NDRG3 gene expressions by GDF15 in bladder carcinoma cells. Interestingly, our results indicated that GDF15 upregulates the NDRG2 in T24 cells but not in HT1376 cells suggesting that the regulation of GDF15 on NDRG2 expression is cell-type specific. Further whole genome profiling assays and the studies of molecular regulatory mechanisms are worthy investigations in determining the downstream genes and the signaling pathways of GDF15 in bladder carcinoma cells.

Epithelial-mesenchymal transition (EMT), a process during which epithelial cells loss epithelial cell markers and gain mesenchymal cell markers, has been regarded as a biomarker of tumor initiation and progression in bladder cancer^29^. The cadherin switch (From E-cadherin to N-cadherin) is a unique feature of EMT and a critical step associated with greater cell invasiveness and poor prognosis of bladder cancer *in vitro* and *in vivo*^30^,^31^,^32^,^33^. The subsequent upregulation of N-cadherin and down-regulation of E-cadherin following GDF15 knockdown in HT1376 cells revealed that GDF15 inhibited the EMT process in bladder carcinoma cells. Since F-actin polarization is the key factor to cell migration^34^, our immunofluoresence stain found that GDF15-knockdown enhanced the staining intensity of F-actin at the leading edge in HT1376 cells suggesting that GDF15 decreases cell invasion may via the downregualtion of EMT and F-actin polarization in bladder carcinoma cells.

In conclusion, this study proves that GDF15 is a p53- and demethylation-upregulated gene. GDF15 inhibited cell growth in bladder carcinoma cells *in vitro* and *in vivo*. GDF15 downregulates cell invasion by blocking the EMT and F-actin polarization. Upregulations of MASPIN, NDRG1, NDRG2, and NDRG3 expressions may account for the antitumor characteristics of GDF15 in human bladder carcinoma cells. Further *in vivo* studies in comparison the GDF15 levels in urine with the GDF15 levels in bladder tissues in order to determine GDF15 as the tumor maker of bladder cancer can be warranted.

## Material and Methods

### Cell culture and chemicals

RT4, HT1376, and T24 cell lines were obtained from the Bioresource Collection and Research Center (BCRC, Taiwan) and maintained as previously described^27^. The identity of cells was confirmed by short tandem repeat (STR-PCR) analysis (Mission Biotech, Taiwan). All experiments were performed between passages 15 to 35. The RT4 cells are p53 wild-type transitional-cell carcinoma cells derived from explants of a recurrent papillary bladder tumor; HT1376 cells are well-differentiated bladder carcinoma cells with tumorigenic activity, which were derived from a grade 3 bladder carcinoma; and T24 cells are poorly differentiated transitional carcinoma cells with low tumorigenic activity^35^,^36^,^37^. We purchased fetal calf serum (FCS) from HyClone (Logan, UT, USA), RPMI 1640 media from Life Technologies (Rockville, MD, USA), recombinant human GDF15 (rhGDF15) from PeproTech (Rocky Hill, NJ, USA), matrigel from BD Biosciences (Bedford, MA, USA), and doxorubicin, camptothecin, and 5′-aza-2′deoxycytidine from Sigma (St. Louis, MO, USA).

### Expression vector constructs and stable transfection

Human GDF15 and p53 expression vectors were constructed as previously described^7^,^38^. Expression vectors were introduced into the HT1376 and the T24 cells by electroporation, and the cells were selected as described previously^27^. The mock-transfected HT1376 (HT-DNA) and T24 (T24-DNA) cells were transfected using a controlled pcDNA3 expression vector (Invitrogen, Carlsbad, CA, USA) and clonally selected in the same manner as described above.

### Knockdown GDF15

HT1376 and RT-4 cells were transducted with Mission® non-target shRNA control transduction particles (SI_SHC002V, Sigma) or GDF15 Mission® shRNA lentiviral transduction particles (SI_NM_004864.1-283S1, Sigma) according to the manufacturer’s protocol. Two days after transduction, the cells (HT-COLsi and HT-GDF15si) were selected by incubation with 10 μg/ml puromycin dihydrochloride.

### Immunoblotting assay

Equal quantities of cell extract were loaded onto a 10% SDS-polyacrylamide gel and analyzed using the Western lightning plus-ECL detection system (PerkinElmer, Inc, Waltham, MA, USA). Blotting membranes were probed using GDF15 antiserum (A0185; Abclonal, Cambridge, MA, USA), MASPIN antiserum (554292; BD Biosciences), NDRG1 antiserum (42-6200; Invitrogen), NDRG2 antiserum (ab169775; Abcam, Cambridge, MA, USA), NDRG3 antiserum (ab131266; Abcam), E-cadherin antiserum (1.B.54; Santa Cruz Biotechnology), N-cadherin antiserum (AJ1526a; Abgent, San Diego, CA, USA), SLUG antiserum (C19G7; Cell signaling, Danvers, MA, USA), SNAIL antiserum (ab117866; Abcam), or β-actin antiserum (I-19, Santa Cruz Biotechnology). Band intensities were recorded using the Chemi Genius II BioImaging System of Syngene (Cambridge, UK) and analyzed using the GeneTool Program of ChemiGenius (Syngene).

### Real-time reverse transcription-polymerase chain reaction (RT-qPCR)

Total RNA was isolated using Trizol reagent and cDNA was synthesized using the superscript III preamplification system as previously described^39^. Real-time polymerase chain reactions (qPCR) were performed using an ABI StepOne Plus Real-Time PCR system (Applied Biosystems, Foster City, CA, USA). FAM dye-labeled TaqMan MGB probes as well as PCR primers for human GDF15 (Hs00171132_m1), NDRG1 (Hs00608387_m1), NDRG2 (Hs01045115_m1), NDRG3 (Hs00223890_m1), MASPIN (Hs00985283_m1), E-cadherin (Hs00170423_m1), N-cadherin (Hs00983062_m1), and β-actin (Hs01060665_g1) were purchased from Applied Biosystems.

### Cell Proliferation Assays

Cell proliferation was measured by ^3^H-thymidine incorporation assay as previously described^40^, or measured with MTS assay kit (Promega Biosciences, San Luis Obispo, CA, USA). Cells (2000 cells/well) were grown in 100 μl RPMI 1640 medium with 10% FCS for different periods as indicated. Cells were washed with PBS twice, then incubated with freshly prepared, combined MTS/phenazine methosulfate (ratio of 1:1 by volume) solution for 3 hours at 37 °C in a humidified 5% CO_2_ atmosphere. The absorbance of the formazan product was counted at 490 nm by the ELISA microplate reader (Dynex Technologies, Chantilly, VA, USA).

### Enzyme-linked immunosorbent assay

Cells were incubated in 0.5 ml of RPMI 1640 medium supplemented with 10% (v/v) FCS and various drugs as indicated. Following 24 hours of incubation, conditioned media were collected for a GDF15 enzyme-linked immunosorbent assay as previously described^7^. GDF15 level in each sample was adjusted according to the concentration of protein measured in a whole cell extract using a bicinchoninic acid protein assay kit (Pierce Biotechnology, Rockford, IL, USA).

### Matrigel invasion assay

The matrigel invasion assay was performed as previously described^41^. Cells that migrated to the matrigel-coated transmembrane were fixed in 4% (w/v) paraformaldehyde and stained with 0.1% (w/v) crystal violet solution for 30 minutes. Images were captured using a digital camera connected to an inverted microscope (IX71, Olympus, Tokyo, Japan) and analyzed using PAX-it Digital Image Management & Image Analysis software following standardization for light intensity.

### F-actin staining

Cells were seeded onto the culture dishes (MatTek, Ashland, MD) that had been precoated with 50 μl of fibronectin. F-actin protein expression was determined with Texas Red X-Phalloidin as previously described^41^. Immunofluorescence was examined using a confocal microscope (LSM510 Meta, Zeiss, Oberkochen, Germany).

### Reporter vector constructs and reporter assay

The human MASPIN and the NDRG1 reporter vectors were constructed as previously described^7^,^40^. The DNA fragment containing the enhancer/promoter of the GDF15 gene (−2887 to +3) was synthesized with primers (5′-GGTACCGACAAGAGCAGATTCATCC-3′ and 5′-TACTCTCCTCCTCCCCTAAC-3′) by PCR using the BAC clone (CTC-251H24; Invitrogen) as target DNA. The DNA fragment was cloned into the pGL3-Basic vector (Promega BioScience) vector at the *Kpn I* and *Xma I* cutting sites. A 6.7 kbp DNA fragment was subtracted from a BAC clone (PR3-469A13; Invitrogen) and cloned into the pGEM-3 vector (Promega BioScience) with the *Hind III* cutting site. The DNA fragment containing the enhancer/promoter of the 5′-flanking region of the NDRG3 gene (−5734 to +178) was cloned into the pGL3-Basic vector at the *Hind III* and *Nco I* cutting sites. Proper ligation and orientation of the reporter vectors were confirmed by extensive restriction mapping and sequencing. Cells were plated onto 24-well plates at a concentration of 10^4^ cells/well for one day prior to transfection. Cells were then transiently transfected and luciferase activity was determined in relative light units (RLU) as described previously^38^.

### Tumor xenograft study

Male nude mice (BALB/cAnN-Foxn1, 4 weeks old) were purchased from the animal center of the National Science Council in Taiwan. Animal studies were performed in accordance with the Guide for Laboratory Animal Facilities and Care (Council of Agriculture Executive, Taiwan) and approved by the Chang Gung University Animal Research Committee (IACUC approval No. CGU08-074). The mice were anesthetized intraperitoneally with 100–150 μl of inoculum (2.5% (v/v) tribomoethanol and 2.5% (v/v) tert-amyl-alchol in Tris buffer solution), and 3 × 10^6^ cells/100 μl cells were injected subcutaneously into one side of the lateral back wall close to the shoulder of each mouse. Xenograft growth was measured by vernier calipers and tumor volume was calculated using a previously described formula: Volume = [π/6 × larger diameter × (smaller diameter)^2^]^40^.

### Statistical analysis

Results are expressed as the mean ± S.E. of at least three independent replications of each experiment. Statistical significance was determined by one-way ANOVA and Student’s *t* test using the SigmaStat program for Window version 2.03 (SPSS Inc, Chicago, IL, USA).

## Additional Information

**How to cite this article**: Tsui, K.-H. *et al.* Growth differentiation factor-15: a p53- and demethylation-upregulating gene represses cell proliferation, invasion, and tumorigenesis in bladder carcinoma cells. *Sci. Rep.* **5**, 12870; doi: 10.1038/srep12870 (2015).

## Supplementary Material

Supplementary Information
