# Supplementary material for: Growth differentiation factor-15: a p53- and demethylation-upregulating gene represses cell proliferation, invasion, and tumorigenesis in bladder carcinoma cells
Source: Sci Rep. 2015 Aug 7;5:12870. doi: 10.1038/srep12870 (PMC4528199; doi:10.1038/srep12870)
Supplement: Supplementary Information [file srep12870-s1.pdf]

Growth differentiation factor-15: a p53- and demethylation-upregulating gene represses cell proliferation, invasion, and tumorigenesis in bladder carcinoma cells

Ke-Hung Tsui[1,3,#], Shu-Yuan Hsu[2,#], Li-Chuan Chung[2], Yu-Hsiang Lin[1], Tsui-Hsia Feng[4], Tzu-Yi Lee[2], Phei-Lang Chang[1,3], & Horng-Heng Juang[2,*]

[1]Department of Urology, Chang Gung Memorial Hospital, Kwei-Shan, Tao-Yuan, Taiwan, ROC
[2]Department of Anatomy, College of Medicine, Chang Gung University, Kwei-Shan, Tao-Yuan, Taiwan, ROC
[3]Department of Traditional Chinese Medicine, College of Medicine, Chang Gung University, Kwei-Shan, Tao-Yuan, Taiwan, ROC
[4]School of Nursing, College of Medicine, Chang Gung University, Kwei-Shan, Tao-Yuan, Taiwan, ROC

Supplemental Figure 1

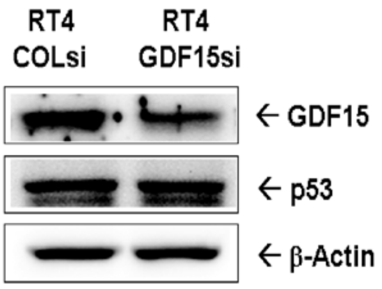

Figure Supplement 1. Modulation of GDF15 on p53 expression in human bladder carcinoma RT4 cells. Expressions of p53, GDF15, and β-actin in mock-knockdown RT4 (RT4-COLsi) and GDF15 knockdown RT4 (RT4-GDF15si) cells were determined by immunoblotting assays.
